# Heat-killed *Prevotella intermedia* promotes the progression of oral squamous cell carcinoma by inhibiting the expression of tumor suppressors and affecting the tumor microenvironment

**DOI:** 10.1186/s40164-024-00500-y

**Published:** 2024-03-21

**Authors:** Yifan Zhou, Yao Qin, Jingjing Ma, Zhiyuan Li, Weiwei Heng, Lei Zhang, Hong Liu, Ruowei Li, Miaomiao Zhang, Qiao Peng, Pei Ye, Ning Duan, Ting Liu, Wenmei Wang, Xiang Wang

**Affiliations:** Nanjing Stomatological Hospital, Affiliated Hospital of Medical School, Institute of Stomatology, Nanjing University, 30 Zhongyang Road, Nanjing, 210008 China

**Keywords:** Oral squamous cell carcinoma, *Prevotella intermedia*, IL-17 signal pathway, GABAergic system, Tumor suppressor genes

## Abstract

**Background:**

Oral microbial dysbiosis contributes to the development of oral squamous cell carcinoma (OSCC). Our previous study showed that *Prevotella intermedia* (*P*. *intermedia*) were enriched in the oral mucosal surface, plaque, and saliva of patients with OSCC. Intratumoral microbiome could reshape the immune system and influence the development of various tumors. However, the invasion status of human OSCC tissues by *P*. *intermedia* and the pathway through which intratumoral *P*. *intermedia* potentiates tumor progression remain unexplored.

**Methods:**

*P*. *intermedia* in human OSCC or normal tissues was detected by FISH. A mouse OSCC cell line SCC7 was adopted to investigate the effects of heat-killed *P*. *intermedia* treatment on cell proliferation, invasion, and cytokine release by using CCK-8 assay, transwell invasion assay and ELISA. Moreover, we established a mouse transplanted tumor model by using SCC7 cells, injected heat-killed *P*. *intermedia* into tumor tissues, and investigated the effects of heat-killed *P*. *intermedia* on tumor growth, invasion, cytokine levels, immune cell infiltrations, and expression levels by using gross observation, H&E staining, ELISA, immunohistochemistry, mRNA sequencing, and transcriptomic analysis.

**Results:**

Our results indicated that* P*. *intermedia* were abundant in OSCC and surrounding muscle tissues. Heat-killed *P*. *intermedia* promoted SCC7 cell proliferation, invasion and proinflammatory cytokine secretions, accelerated transplanted tumor growth in mice, exacerbate muscle and perineural invasion of OSCC, elevated the serum levels of IL-17A, IL-6, TNF-α, IFN-γ, and PD-L1, induced Treg cells M2 type macrophages in mouse transplanted tumors. The data of transcriptomic analysis revealed that heat-killed *P*. *intermedia* increased the expression levels of inflammatory cytokines and chemokines while reduced the expression levels of some tumor suppressor genes in mouse transplanted tumors. Additionally, IL-17 signaling pathway was upregulated whereas GABAergic system was downregulated by heat-killed *P*. *intermedia* treatment.

**Conclusions:**

Taken together, our results suggest that *P*. *intermedia* could inhibit the expression of tumor suppressors, alter the tumor microenvironment, and promote the progression of OSCC.

**Supplementary Information:**

The online version contains supplementary material available at 10.1186/s40164-024-00500-y.

## Introduction

Oral squamous cell carcinoma (OSCC) is one of the most severe and frequent forms of head and neck cancer to date, with a high recurrent rate and low overall survival rate [1]. Although previous studies revealed multiple risk factors for OSCC, the pathophysiological mechanisms are still unclear. In recent years, with the development of detection technology and an in-depth understanding of the tumor microenvironment (TME), increasing evidence has confirmed that microbial dysbiosis in the oral cavity is a potential risk factor for oral cancer [2]. Notably, periodontitis-related pathogens play a critical role in OSCC progression.

In a previous study, we found that *Prevotella intermedia* (*P. intermedia*) was enriched in the profiling saliva, supragingival plaque, and oral lesion surface of OSCC patients [3]. Several studies have indicated that the abundance of *P*. *intermedia* is significantly increased in OSCC sites compared with normal oral mucosa [4, 5]. Furthermore, a study showed that *P*. *intermedia* was positively correlated with an increased risk of oral cancer [6]. Interestingly, *P*. *intermedia* was confirmed to be involved in several cancers, such as colorectal cancer [7, 8], esophageal squamous carcinoma, and oropharyngeal cancer [9]. These findings indicated that *P*. *intermedia* might be a new candidate that promotes the tumor progression of OSCC.

It has been shown that diverse immunological responses, including the production of proinflammatory cytokines, can all be triggered by microbes, affect the TME, inhibit the antitumor immune response, and contribute to the progression of tumors [10]. Several studies have revealed the roles of several oral microbes in shaping the TME and influencing cancer progression via IL-17 signaling, NF-κB signaling, and Toll-like receptor signaling pathways [10–12]. An early study indicated that *P. intermedia* can invade human OSCC cells in vitro [13]. Therefore, the invasion status of human OSCC tissues by *P. intermedia* and the pathway through which *P*. *intermedia* potentiates tumor progression were explored in the present study.

**Fig. 1:**
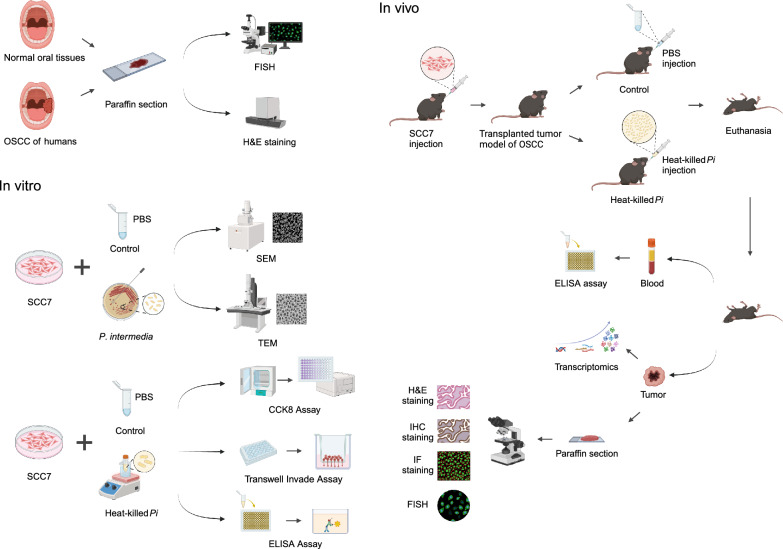
Flow chart of the experiments. *P*. *intermedia* detection in OSCC and normal tissues, in vitro study of SCC7 cells, and in vivo study in a murine transplanted tumor model of OSCC

In this study, we aimed to investigate the effects of heat-killed *P*. *intermedia* on OSCC progression and the underlying mechanisms. Firstly, we explored the invasion status of human OSCC tissues by *P. intermedia* through observing and comparing the quantity and distribution of *P*. *intermedia* in normal oral mucosa and OSCC tissues of humans. Subsequently, we performed in vitro studies by using a mouse OSCC cell line SCC7 and investigated the effects of heat-killed *P*. *intermedia* treatment on cell proliferation, invasion, and cytokine release. Moreover, we established a mouse transplanted tumor model using SCC7 cells, injected heat-killed *P*. *intermedia* into tumor tissues, and investigated the effects of heat-killed *P*. *intermedia* on tumor growth, invasion, cytokine levels, immune cell infiltrations, and gene expressions (Fig. 1). Our study may build an evidence base to further explore the roles of *P*. *intermedia* and other oral pathogens in OSCC progression and provide insights for the development of new potential treatments that target pathogens in the future.

## Materials and methods

### Clinical samples

The resected tumor tissues (n = 15) from OSCC patients and normal tissues (n = 10) from patients who underwent orthognathic surgery were obtained from Nanjing Stomatological Hospital, Affiliated Hospital of Medical School, Nanjing University. These patients who underwent orthognathic surgery have good oral hygiene and no confirmed periodontal diseases were found through periodontal examination. Sterile surgical samples were freshly collected and formalin-fixed and paraffin-embedded for further hematoxylin–eosin staining (H&E) and fluorescent in situ hybridization (FISH) validation. We obtained written informed consent from all patients. The study protocol was approved by the Ethics Committee of Nanjing Stomatological Hospital, Affiliated Hospital of Medical School, Nanjing University (IRB approval number: 2018NL-008 (KS) & NJSH-2023NL-18) and the Animal Ethical and Welfare Committee of Nanjing University (IACUC-D2202108 & IACUC D2303084).

### Fluorescent in situ hybridization (FISH)

Spectrum-Yellow FISH labeled with RNAscope^™^ Probe-B-*P*. *intermedia*-16SrRNA-C1 (ACD, 1197771-C1, GCCTA-ATACCCGATG-TTGTCCACAT-ATGGCATCTG-ACGTGGACC) was performed according to the manufacturer’s instructions. The specific probe detecting* P*. *intermedia* was designed and developed based on the NCBI database (Additional file 1: Fig S1). Fluorescence microscopic analysis was conducted with a confocal microscope. The Panoramic 250 Flash system (3DHISTECH) was used to scan the slides.

### Bacterial strain

*P*. *intermedia* strain ATCC 25611 was purchased from BeNa Culture Collection (Beijing, China) and grown in Columbia blood agar plates under anaerobic conditions at 37 °C for 3–4 days. The monoclonal colonies were then picked up and inoculated in sterilized brain heart infusion broth (BHI) supplemented with yeast extract (1 mg/mL), hemin (5 μg/mL), and vitamin K (1 μg/mL) until the cells reached the logarithmic growth phase.

### Cell culture

The mouse OSCC cell line SCC7 (Cellcook Biotech Co., Ltd. Guangzhou, China) was cultured at 37 °C with 5% CO_2_ in Roswell Park Memorial Institute (RPMI) 1640 medium including 10% fetal bovine serum (FBS) and 1% penicillin (1 × 105 U/L)-streptomycin (100 mg/L) solution (PS).

### Transmission electron microscopy (TEM)

A transmission electron microscope (TEM) was used to visualize the morphology of intracellular bacteria. SCC7 cells were seeded with complete cell culture medium (1640 medium with 10% FBS and 1% PS). Before *P*. *intermedia* invasion, the cells were washed twice with phosphate-buffered saline (PBS), and antibiotic-free medium was added. *P*. *intermedia* was collected during the logarithmic phase, washed twice with PBS, and added to the cells at an MOI of 10, and the cells were incubated in the cell incubator for 3 h. After infection, the cells were washed with PBS 3 times, and 2.5% glutaraldehyde (Sigma‒Aldrich) was added for 5 min at 25 °C protected from light. All cells were observed using a Hitachi electronic microscope at 80 kV.

### Scanning electron microscopy (SEM)

SCC7 cells were added to slides and allowed to adhere overnight. The infection steps were the same as described for TEM. After 3 h, the cells were fixed with 2.5% glutaraldehyde (Sigma‒Aldrich) in PBS. The cells were dehydrated with a gradient of increasing alcohol concentrations (40–120%). Then, they were dried using the critical point method (Quorum, Model K 850). The samples were gold-sputtered (Hitachi, MC1000) to generate conductivity and then coated with iridium fast coating (5 nm) on a Leica EMACE600 and imaged on a Hitachi SU8100 field-emission SEM.

### Cell proliferation assay

Cell Counting Kit‐8 (CCK‐8) assays (Dojindo Molecular Technologies, XiongBen, Japan) were used to assess the proliferation ability of SCC7 cells. SCC7 cells were seeded into 96‐well plates at a concentration of 3 × 10^3^ cells each well in 100 μl of medium, and after adherence overnight, we treated the cells according to the experimental design. Heat-killed *P*. *intermedia* (1, 10, 50, 100 MOIs) was added to SCC7 cells for 24, 48, 72 h, respectively. There were 5 replicate wells in each group. Afterward, 10 µL of CCK‐8 solution was added to each well of the plate. After 2 h of incubation at 37 °C with 5% CO_2_, the absorbance was measured at 450 nm by a microplate reader. OD values were analyzed as the mean ± standard deviation.

### Cell invasion assay

Transwell upper chambers (8-μm pore size; Corning Incorporated, Toledo, NY, USA) were pre-coated with Matrigel (Corning Incorporated). 3 × 10^4^ SCC7 cells were suspended in 200 μL medium and then plated on the upper chambers with Matrigel. Then, 10% FBS was added in 500 μL medium, which was prepared onto the lower chambers. After culture for 24 h, the invaded SCC7 cells on the lower surface of the chamber were fixed by paraformaldehyde solution for 30 min at room temperature and stained using 0.1% crystal violet (Solarbio) for 30 min. Finally, the number of cells on the lower surface of the membrane were observed and counted using a microscope (Olympus) in five random fields.

### Enzyme-linked immunosorbent assay (ELISA)

Analysis was performed on cell culture supernatants or serum to determine the concentrations of cytokines, including IL-17A, IL-6, IL-10, IL-1β, PD-L1, IFN-γ and TNF-α. All ELISA kits were purchased from Multiscience Biotechnology Co., Ltd. (Hangzhou, Zhejiang, China). SCC7 cells treated with heat-killed *P*. *intermedia* for 12 h were cultured using the abovementioned method and grouped according to the MOIs of 1, 10, 50, and 100.

### Murine transplanted tumor model of OSCC

C57BL/6 J mice (6 weeks old, female) were housed in a stable environment with a 12:12 h light/dark cycle and had free access to food and water. The humidity was kept between 55 and 65% and the temperature was maintained between 23% and 25 °C. To eliminate interference from other bacteria in the oral cavity, mixed antibiotics containing metronidazole (1 mg/ml; Sigma‒Aldrich), neomycin (1 mg/ml; MCE), ampicillin (1 mg/ml; MCE), and vancomycin (0.5 mg/ml; MCE) were administered in drinking water for 3 days before injection. SCC7 cells resuspended in PBS (3 × 10^5^, 5 μl/per) were submucosally injected into the buccal mucosa to establish a OSCC transplanted tumor model [14]. *P*. *intermedia* strain ATCC 25611 was collected during the logarithmic phase, washed twice with PBS, inactivated in an 80 °C water bath for 2 h, and diluted with PBS (3 × 10^2^ CFU, 5 μl/per). The experimental mice were divided randomly into the control group and heat-killed *P*. *intermedia* group (6 mice per group). All mice received submucosal injection of SCC7 suspension liquid (3 × 10^5^, 5 μl/per). Each mouse in the heat-killed *P*. *intermedia* group was injected with heat-killed *P*. *intermedia* (3 × 10^2^ CFU, 5 μl/per) into the palpable tumor 7 days after SCC7 cell injection. Tumor volume and body weight were measured at 2 day intervals until the end of the experiment. Tumor volume = maximum diameter × vertical diameters^^2^/2. Fourteen days after the first injection, the mice were sacrificed for serum and tumor quantification. All animal experiments were performed in compliance with ethical regulations.

### Hematoxylin and eosin staining, immunohistochemistry, and immunofluorescence

Mouse tumor specimens were formalin-fixed, dehydrated, paraffin-embedded and cut into 5 μm sections using routine protocols for staining with H&E. Images were captured by microscopy (Olympus CX23; Olympus Corporation, Tokyo, Japan). The Panoramic 250 Flash system (3DHISTECH) was used to scan the slides.

Immunohistochemistry was processed in the same way as described above and then stained with IL-17A (Abcam, ab91649), IL-23 (NOVUS BIO, NBP1-76697), IL-6 (Abcam, ab290735), KI-67 (Abcam, ab237728), matrix metalloproteinase-9 (MMP-9) (Abcam, ab283575), GABBR2 (Abcam, ab75838), FASL (ABclonal, A0234), P63 (ABclonal, A19652), CD8α (Abcam, ab217344), FOXP3 (CST, 98377S), F4/80 (Abcam, ab6640), CD206 (Abcam, ab64693) and DAPI (Abcam, ab104139). Images were taken using a microscope or a confocal laser scanning microscope. The panoramic 250 Flash system (3DHISTECH) was used to scan the slides. All immunohistochemistry assays included negative controls and positive controls. The IHC-positive areas were stained brown in the cytoplasm, and the average optical density (AOD) [AOD = Integrated Optical Density (IOD) SUM/Area SUM] was measured using ImageJ (IHC toolbox plugin). The relative AOD values were calculated using the normalization method. At least three random fields of each slide were selected.

### Tumor RNA extraction, mRNA sequencing, and transcriptome analysis

Total RNA was extracted using TRIzol reagent (Invitrogen, CA, USA) according to the manufacturer’s protocol. RNA purity and quantification were evaluated using a NanoDrop 2000 spectrophotometer (Thermo Scientific, USA). RNA integrity was assessed using an Agilent 2100 Bioanalyzer (Agilent Technologies, Santa Clara, CA, USA). Then, the libraries were constructed using the VAHTS Universal V6 RNA-seq Library Prep Kit according to the manufacturer’s instructions. Transcriptome sequencing and analysis were conducted by OE Biotech Co., Ltd. (Shanghai, China).

Differential expression analysis was performed using DESeq25. A Q value < 0.05 and fold change > 2 or fold change < 0.5 were set as the thresholds for significantly differentially expressed genes (DEGs). Hierarchical cluster analysis of DEGs was performed using R (v 3.2.0) to demonstrate the expression pattern of genes in different groups and samples. The radar map of the top 30 genes was drawn to show the expression of upregulated or downregulated DEGs using the R packet ggradar.

Based on the hypergeometric distribution, GO, KEGG pathway, Reactome and WikiPathways enrichment analyses of DEGs were performed to screen the significantly enriched terms using R (v 3.2.0). R (v 3.2.0) was used to draw the column diagram, chord diagram and bubble plot of the significantly enriched terms.

Gene Set Enrichment Analysis (GSEA) was performed using GSEA software8-9. The analysis used a predefined gene set, and the genes were ranked according to the degree of differential expression in the two types of samples. Then, we tested whether the predefined gene set was enriched at the top or bottom of the ranking list.

### Statistical analysis

SPSS 20.0 (IBM, NY, USA) was used for data analysis. ImageJ and GraphPad Prism 9.0 (Graph Software Inc.) were used for image analysis. Data are expressed as the mean ± SD. The difference in measurement data between two groups was assessed using a two-tailed/one-tailed *Student’s t* test. Multiple group comparisons were conducted using one- or two-way analysis of variance (ANOVA). The level of statistical significance was set at p < 0.05.

## Results

### *P*.* intermedia* was abundant in OSCC tissues and invasive to SCC7 cells

**Fig. 2:**
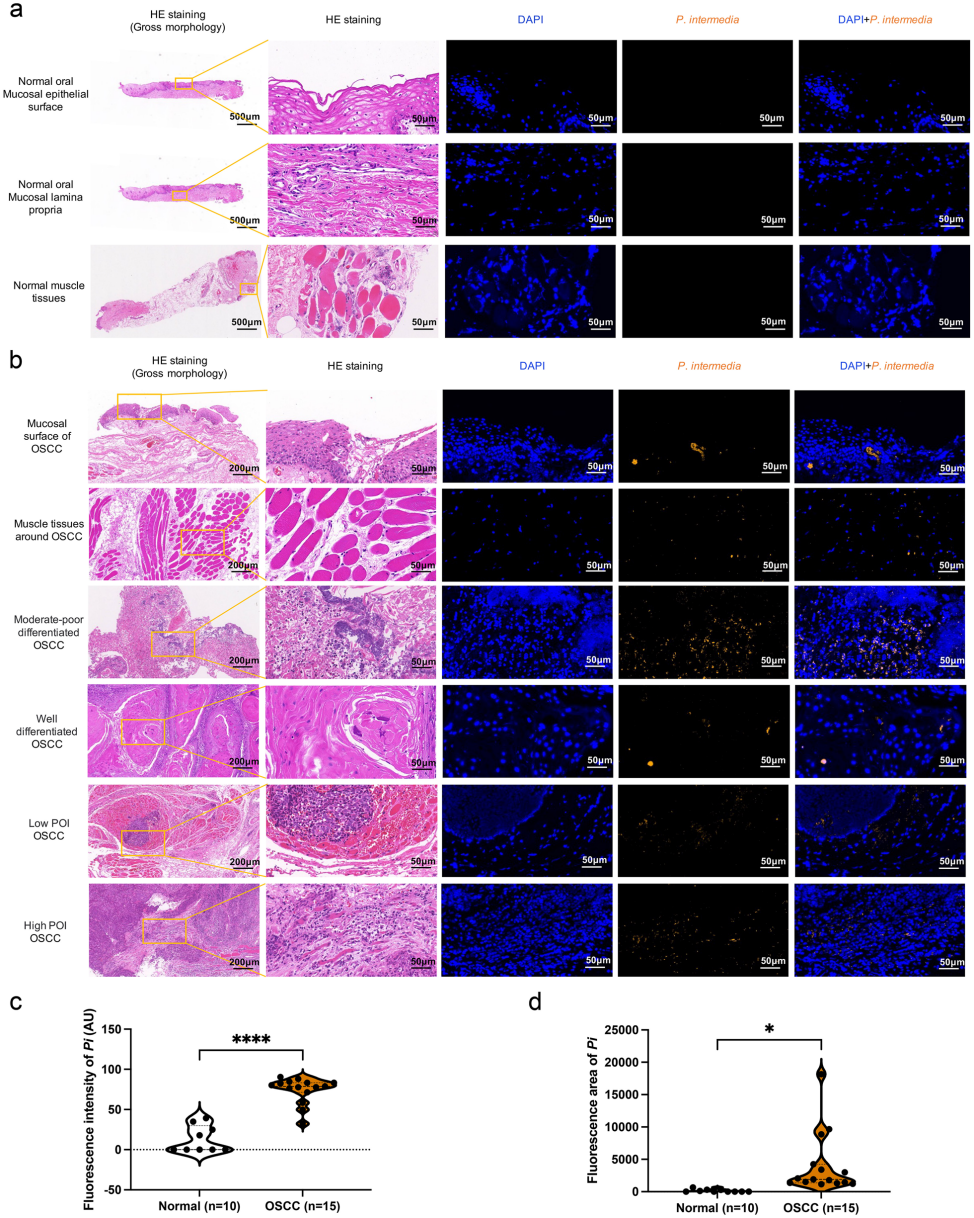
Representative images of H&E and FISH staining of human normal or OSCC tissues. DAPI (blue). *P*. *intermedia* labeled with specific probes (yellow). **a** Normal oral mucosal epithelial surface, mucosal lamina propria, and muscle tissues. **b** Mucosal surface of OSCC, muscle tissues around OSCC, various differentiated OSCC tissues, low or high pattern of invasion (POI) OSCC tissues. **c** Fluorescence intensity difference of *P*. *intermedia* between normal and OSCC tissues. **d** Fluorescence area difference of *P*. *intermedia* between normal and OSCC tissues. Data were analyzed by *t* test. *P < 0.05, ****P < 0.0001

In a previous study, we found that *P*. *intermedia* was enriched in the profiling saliva, gingival plaque, and lesional surface of OSCC patients [3]. To further confirm the intratumoral location of *P*. *intermedia*, we performed an RNA FISH assay on the tumor samples from patients with OSCC using *P*. *intermedia*-specific probes. Simultaneously, we performed the same assay on the normal tissues of healthy individuals. The results showed that *P*. *intermedia* was significantly enriched in OSCC tissues compared to normal tissues. For normal oral mucosa, weak fluorescence signals of *P*. *intermedia* were occasionally detected on the mucosal surface, while no fluorescence signal of* P*. *intermedia* was found in the lamina propria and muscle tissues. For OSCC tissues, however, strong fluorescence signals of *P*. *intermedia* could be commonly detected on the tumor surface, within the tumor tissues, even in the muscle tissues around OSCC. Notably, the fluorescence signals of *P*. *intermedia* in the moderate-poor differentiated OSCC tissues were stronger than those in the well differentiated OSCC tissues. The Pattern of Invasion (POI) of OSCC cells into adjacent tissues can be divided into low-invasiveness and high-invasiveness [15]. Similarly, the fluorescence signals of *P*. *intermedia* in the OSCC tissues with high POI were stronger than those in the OSCC tissues with low POI. The fluorescence intensity and area of *P*. *intermedia*-specific probes were significantly enhanced in OSCC tissues compared with the normal oral mucosal tissues, respectively (*P* < 0.05) (Fig. 2). Therefore, our results indicated that *P*. *intermedia* can clearly invade into OSCC tissues but not into normal oral mucosa.

**Fig. 3:**
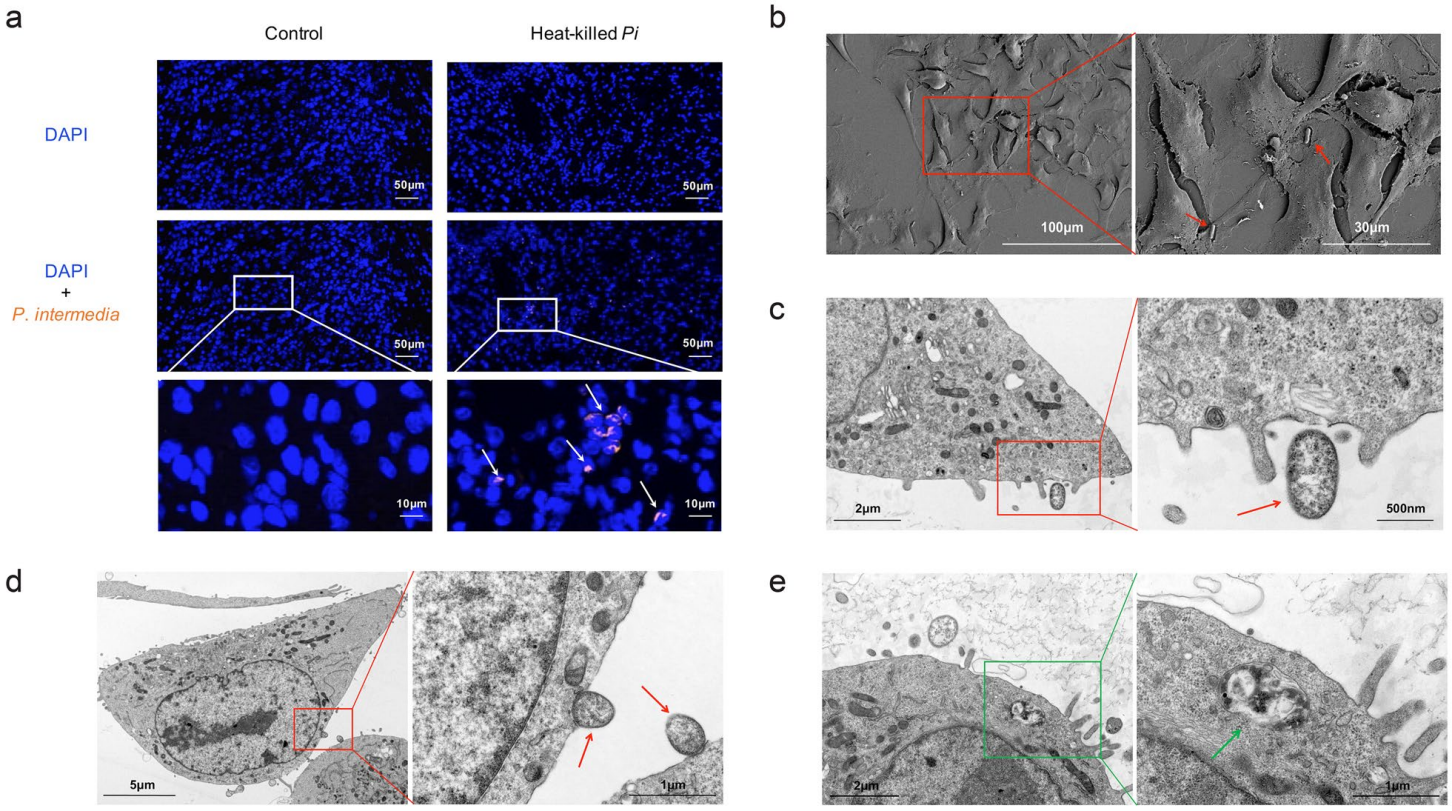
Measurement of *P. intermedia* in mouse transplanted tumors and observation of *P. intermedia*-SCC7 cell cocultures. **a** FISH images of *P*. *intermedia* in OSCC tissues of mouse transplanted tumors using *P*. *intermedia*-specific probes. **b** SEM images of *P. intermedia*-SCC7 coculture for 40 min. **c** TEM images of *P. intermedia*-SCC7 coculture for 40 min. **d** TEM images of *P. intermedia*-SCC7 coculture for 3 h. **e** TEM images of *P. intermedia* in SCC7 cells following *P*. *intermedia*-SCC7 coculture for 3 h. White arrows indicate *P*. *intermedia* in OSCC tissues of mice. Red arrows indicate *P*. *intermedia* that is adhering to SCC7 cells. A green arrow indicates mitotic structures of *P*. *intermedia* within the SCC7 cytosol

Subsequently, in the OSCC tissues of murine transplanted tumor models, *P*. *intermedia* could be detected intercellularly and intracellularly in the heat-killed *P*. *intermedia* group by specific probes (Fig. 3a). To determine whether *P*. *intermedia* ATCC 25611 could invade OSCC cells, we first performed SEM and TEM to observe *P*. *intermedia*-treated SCC7 cells. SEM imaging visualized the migration, adhesion, and invasion of *P*. *intermedia* in SCC7 cells (Fig. 3b). Interestingly, TEM imaging further demonstrated the intracellular localization of *P*. *intermedia* within SCC7 cells (Fig. 3c–e). In summary, our in vivo and in vitro studies indicated that *P*. *intermedia* is invasive to OSCC cells, determining the existence of *P*. *intermedia* infection in OSCC tissues.

### Effects of heat-killed *P*. *intermedia* on SCC7 cell proliferation and invasion at different MOIs in vitro

**Fig. 4:**
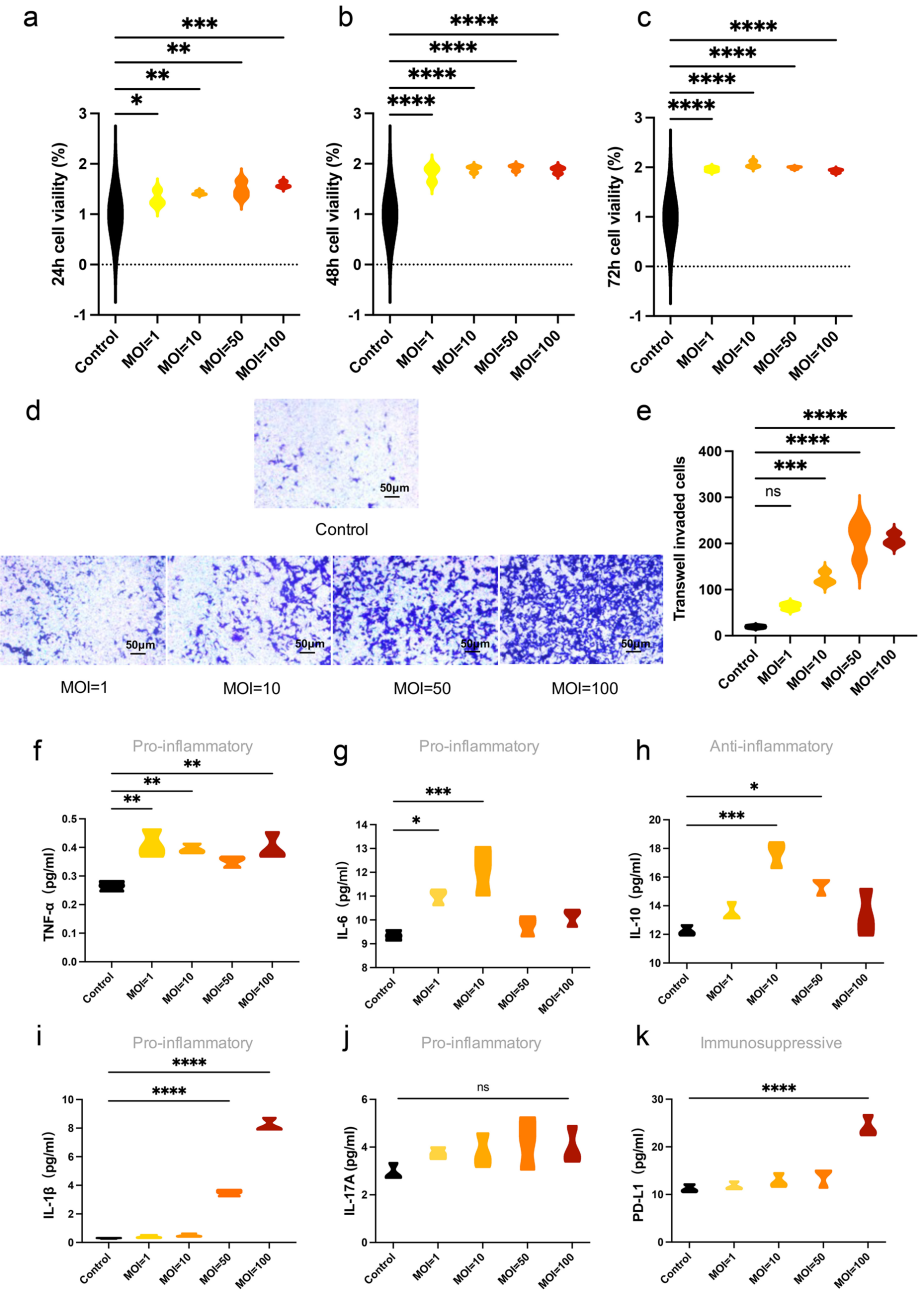
Effects of heat-killed *P*. *intermedia* on SCC7 cell proliferation and cytokine release at different MOIs. **a**–**c** Viability of SCC7 cells treated with/without heat-killed *P*. *intermedia*. **a** Cell viability at 24 h. **b** Cell viability at 48 h. **c** Cell viability at 72 h. **d**, **e** Cell invasion ability at 24 h detected by using transwell assays. **f**–**k** Secretory levels of TNF-α, IL-6, IL-10, IL-1β, IL-17A, and PD-L1 by SCC7 cells treated with/without heat-killed *P*. *intermedia*. Data are the means of values from three independent experiments, shown as the mean ± SEM, and were analyzed using one-way ANOVA. *P < 0.05, **P < 0.01, ***P < 0.001, ****P < 0.0001. ns, not significant.

Since *P. intermedia* could be detected in OSCC tissues and SCC7 cells, we initially investigated whether *P*. *intermedia* is involved in OSCC cell proliferation by treating SCC7 cells with heat-killed *P*. *intermedia*. Among the control group and heat-killed *P*. *intermedia* groups with different MOIs, there was significant difference in cell viability after 24, 48, and 72 h of treatment. The cell viability of the groups treated with heat-killed *P*. *intermedia* at MOIs of 1, 50, and 100 significantly increased compared with that of the control group (*P* < 0.05). As our results showed, heat-killed *P*. *intermedia* effectively promoted the proliferation of SCC7 cells after 24, 48 and 72 h of treatment (Fig. 4a–c). Moreover, the invasive ability of SCC7 cells was significantly triggered after 24 h of treatment with heat-killed *P*. *intermedia* at MOIs of 10, 50, and 100 (*P* < 0.05) (Fig. 4d, e).

### Effects of heat-killed *P*. *intermedia* on the secretory cytokine levels of SCC7 cells in vitro

Dysregulated immune and chronic inflammation are known to be crucially involved in the development of OSCC. To reveal the immune-altering and inflammation-promoting effects of *P*. *intermedia,* we analyzed the cytokine levels in the culture supernatant of SCC7 cells with or without heat-killed *P*. *intermedia* treatment using ELISA. The levels of inflammatory or immune-related cytokines in the heat-killed *P*. *intermedia* group were significantly increased at different MOIs compared with those in the control group (Fig. 4f–k). Specifically, there was a remarkable rise in TNF-α and IL-6 levels following treatment with heat-killed *P*. *intermedia* at an MOI of 1 (*P* < 0.05). There was a significant increase in TNF-α, IL-6 and IL-10 levels at an MOI of 10 (*P* < 0.01). Moreover, there was a marked enhancement in IL-10 and IL-1β levels at an MOI of 50 (*P* < 0.05). Furthermore, there was a significant enhancement in TNF-α, IL-1β and PD-L1 levels at an MOI of 100 (*P* < 0.01).

### Effects of heat-killed *P*. *intermedia* on transplanted tumor growth in mice

**Fig. 5:**
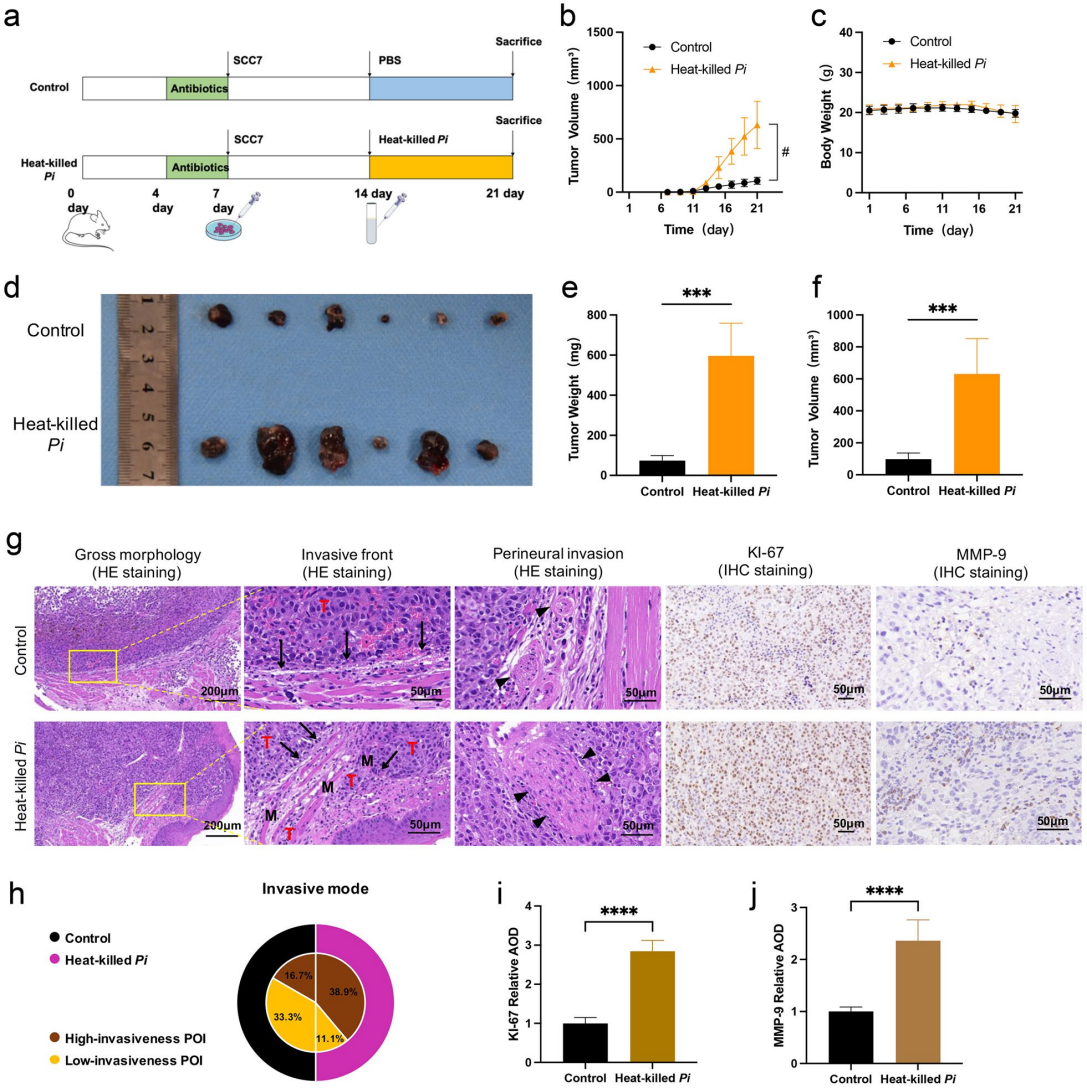
Heat-killed* P*. *intermedia* accelerated transplanted tumor growth in mice. **a** Flow chart of transplanted tumor experiments. **b** Tumor volume changes in mice. **c** Body weight changes in mice. **d** Gross morphology of dissected tumors in each group. **e** Differences in tumor weight between the heat-killed *P*. *intermedia* and control groups. **f** Differences in tumor volume between the heat-killed *P*. *intermedia* and control groups. **g** Invasive fronts (black arrows), muscle (M) and perineural (closed arrowheads) invasion of OSCC tissues (T) are shown according to H&E staining, KI-67 and MMP-9 expressions were determined by IHC staining. **h** Invasive mode comparison between the heat-killed *P*. *intermedia* and control groups. **i**, **j** The relative AOD of KI-67 and MMP-9 IHC staining was compared between the heat-killed *P*. *intermedia* and control groups. Data were analyzed by *t* test. ^#^*P* < 0.05, ***P < 0.001, ****P < 0.0001

To further investigate the effects of *P*. *intermedia* on OSCC progression in vivo, we established a murine transplanted tumor model by submucosally injecting SCC7 cells and subsequently performing heat-killed *P*. *intermedia* injection in the transplanted tumors. After the 21-d follow-up, there was no significant difference in the body weight of mice between the control group and the heat-killed *P*. *intermedia* group (Fig. 5c). Interestingly, the transplanted tumors originating from SCC7 cells in the heat-killed *P*. *intermedia* group displayed significantly enhanced growth in vivo compared with those in the control group (Fig. 5b, d–f). Moreover, the results of IHC staining showed that there was significantly augmented staining of Ki67 in the OSCC tissues of the heat-killed *P*. *intermedia* group compared with that in the control group (Fig. 5g, i). Moreover, inflammatory immune cell infiltration of OSCC tissues were determined according to H&E staining (Fig. 5g). The Pattern of Invasion (POI) of OSCC cells into adjacent tissues can be divided into low-invasiveness and high-invasiveness [15]. Clearly, in the present study, immune cell infiltration with a high-invasiveness POI could be seen in the heat-killed *P*. *intermedia* group, while a low-invasiveness POI could be seen in the control group (Fig. 5g, h). Notably, the results of IHC staining indicated that there was significantly enhanced staining of MMP-9, a typical invasion marker, in the OSCC tissues of the heat-killed *P*. *intermedia* group compared with that in the control group (Fig. 5g, j).

### Heat-killed *P*. *intermedia* changed the serum cytokine levels of transplanted tumor mice

**Fig. 6:**
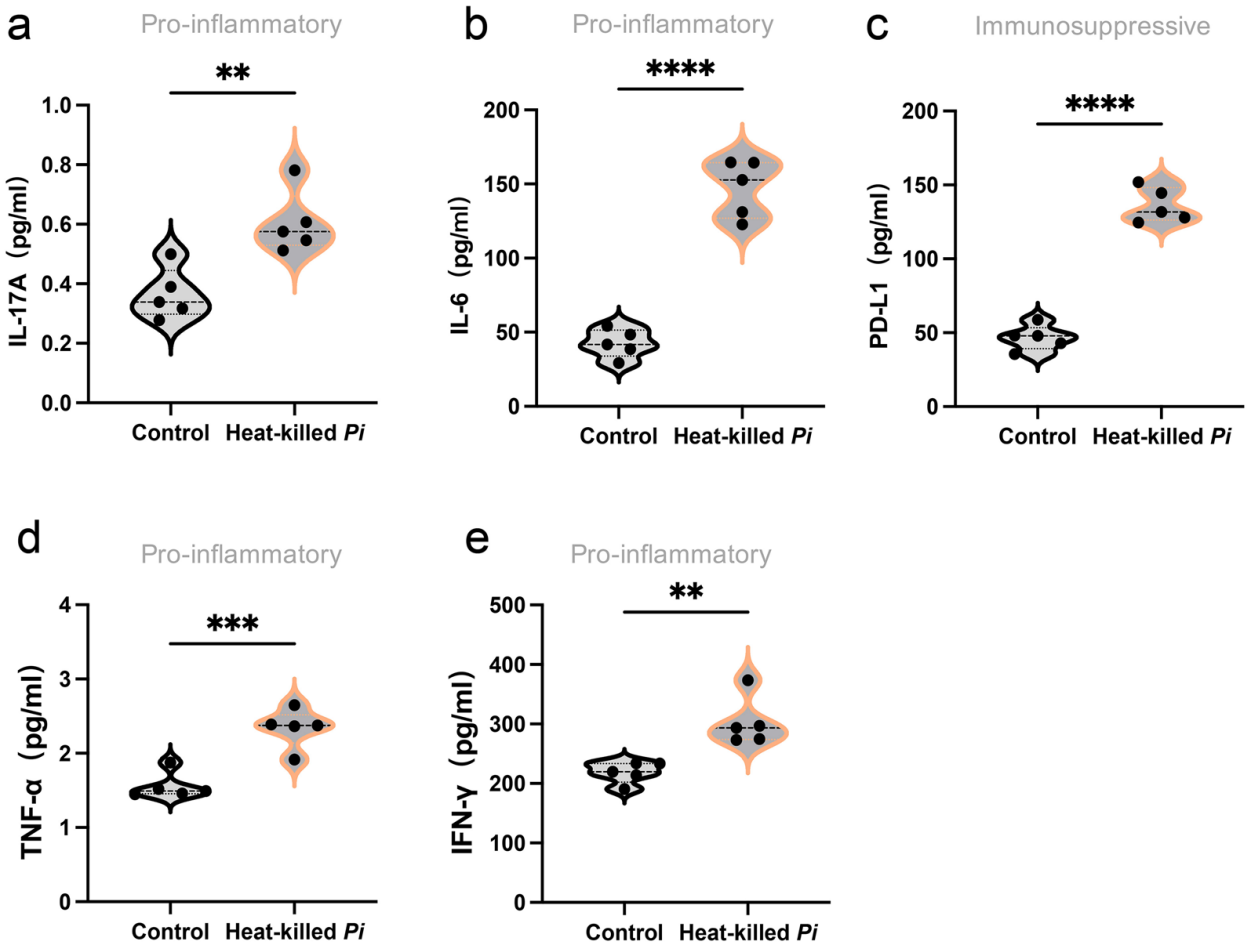
Altered serum cytokine levels by heat-killed *P*. *intermedia* in mice with transplanted tumors. The data are expressed as the mean ± SD and were analyzed using a *t* test. **P < 0.01, ***P < 0.001, ****P < 0.0001

The serum levels of cytokines in the heat-killed *P*. *intermedia* group and control group were examined by using ELISA. Our results showed that heat-killed *P*. *intermedia* significantly enhanced the serum levels of IL-17A, IL-6, PD-L1, TNF-α, and IFN-γ compared with those of the control group (Fig. 6a–e). Based on the above results, *P*. *intermedia* could lead to imbalanced levels of inflammatory cytokines, contributing to further exacerbation of the inflammatory TME.

### Heat-killed *P*. *intermedia* activated IL-17 signaling, inhibited the GABAergic system and extensively inhibited the expression of tumor suppressor genes in mouse transplanted tumors

**Fig. 7:**
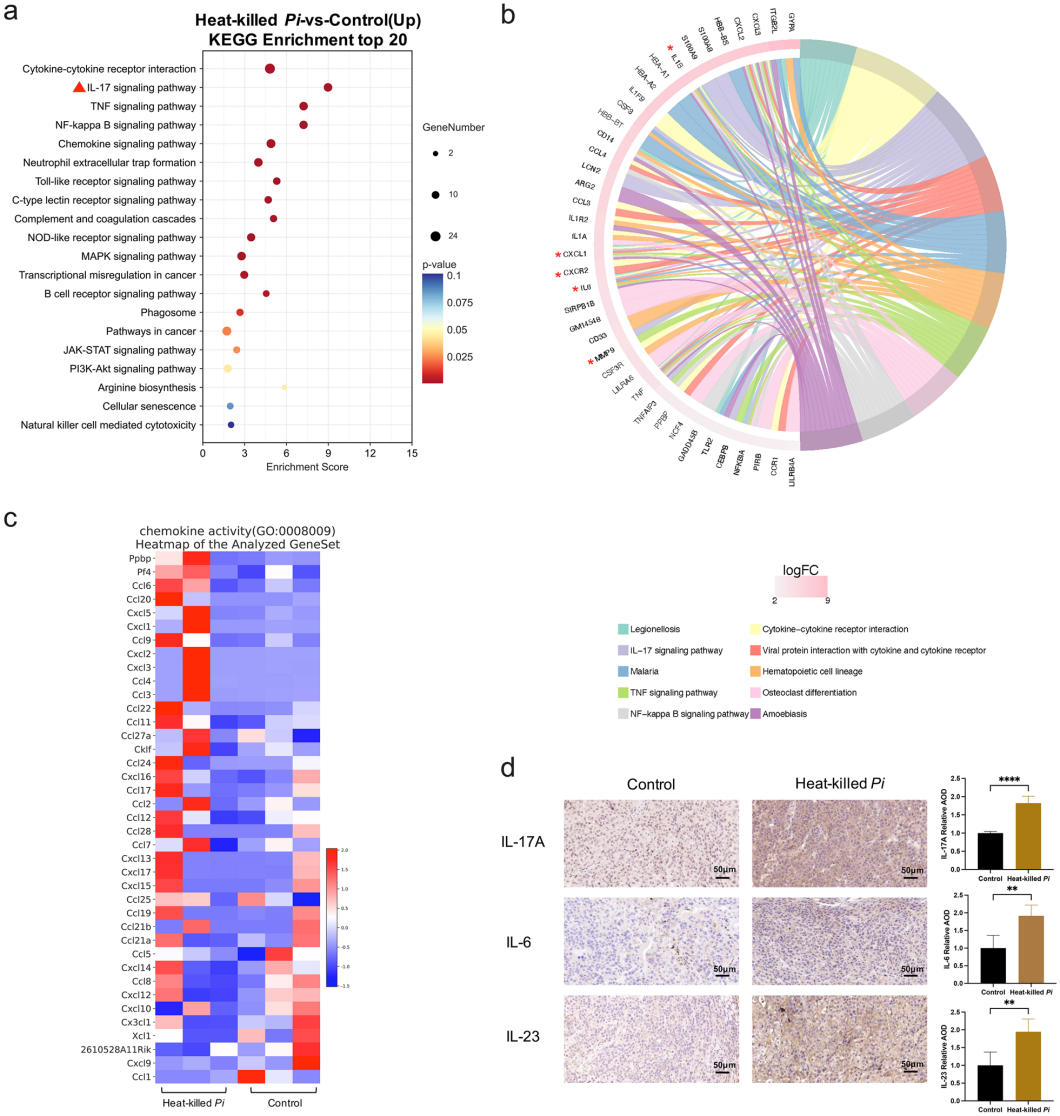
Effects of heat-killed *P*. *intermedia* on the transcriptomics of proinflammatory cytokines and chemokines in transplanted tumor model mice. **a** Top 20 upregulated pathways in the heat-killed *P*. *intermedia* group by using KEGG pathway enrichment analysis. The red triangle indicates OSCC-related top 1 proinflammatory signaling pathways. **b** Chord diagrams of upregulated genes and pathways. Red asterisks indicate important genes in the upregulated signaling pathway. **c** Heatmap of the chemokine activity pathway gene set in both groups. **d** Representative images of IL-17A, IL-6, and IL-23 IHC staining. The relative AOD of IHC staining was compared between the control and heat-killed *P*. *intermedia* groups. The data are shown as the mean ± SD and were analyzed by *t* test. **P < 0.01, ****P < 0.0001

**Fig. 8:**
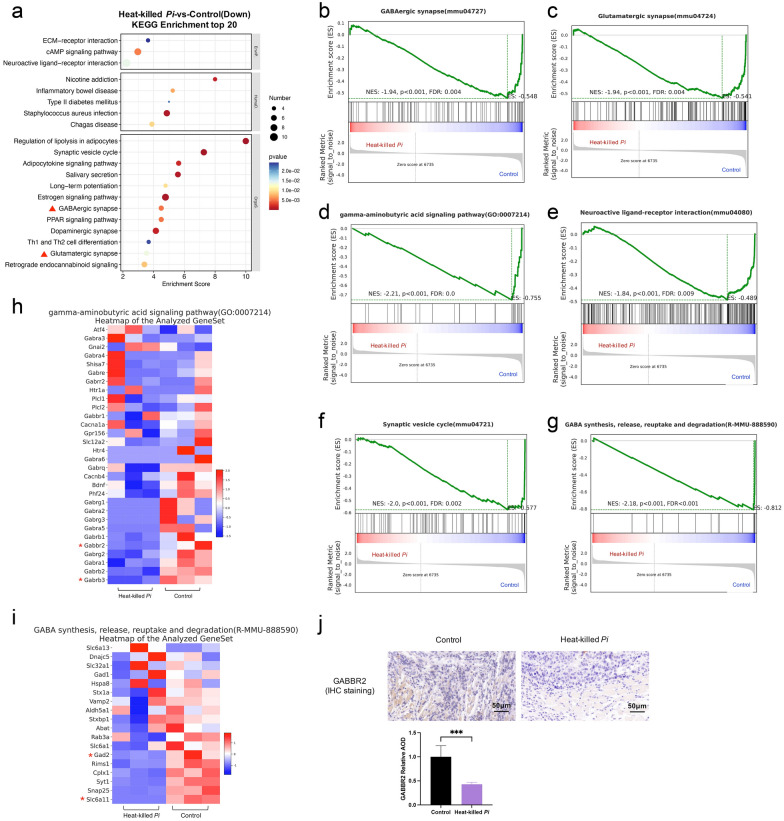
Effects of heat-killed *P*. *intermedia* on the transcriptomics of GABA signaling and GABAergic synapses in transplanted tumor model mice. **a** Top 20 downregulated pathways between the heat-killed *P*. *intermedia* and control groups in KEGG pathway enrichment analysis. Red triangles indicate the GABA-related signaling pathway. **b**–**g** GSEA showing the downregulation in the heat-killed *P*. *intermedia* group. **b** GABAergic synapse downregulation. **c** Glutamatergic synapse downregulation. **d** GABA signaling pathway downregulation. **e** Neuroactive ligand‒receptor interaction downregulation. **f** Synaptic vesicle cycle downregulation. **g** GABA synthesis, release, reuptake and degradation downregulation. **h** Heatmap of the GABA synthesis, release, reuptake and degradation gene set. **i** Heatmap of the GABA signaling pathway gene set. Red asterisks indicate important genes in the GABA-related signaling pathway. **j** Representative images of GABBR2 IHC staining. The relative AOD of GABBR2 IHC staining was compared between the control and heat-killed *P*. *intermedia* groups. The data are shown as the mean ± SD and were analyzed by *t* test. ***P < 0.001

**Fig. 9:**
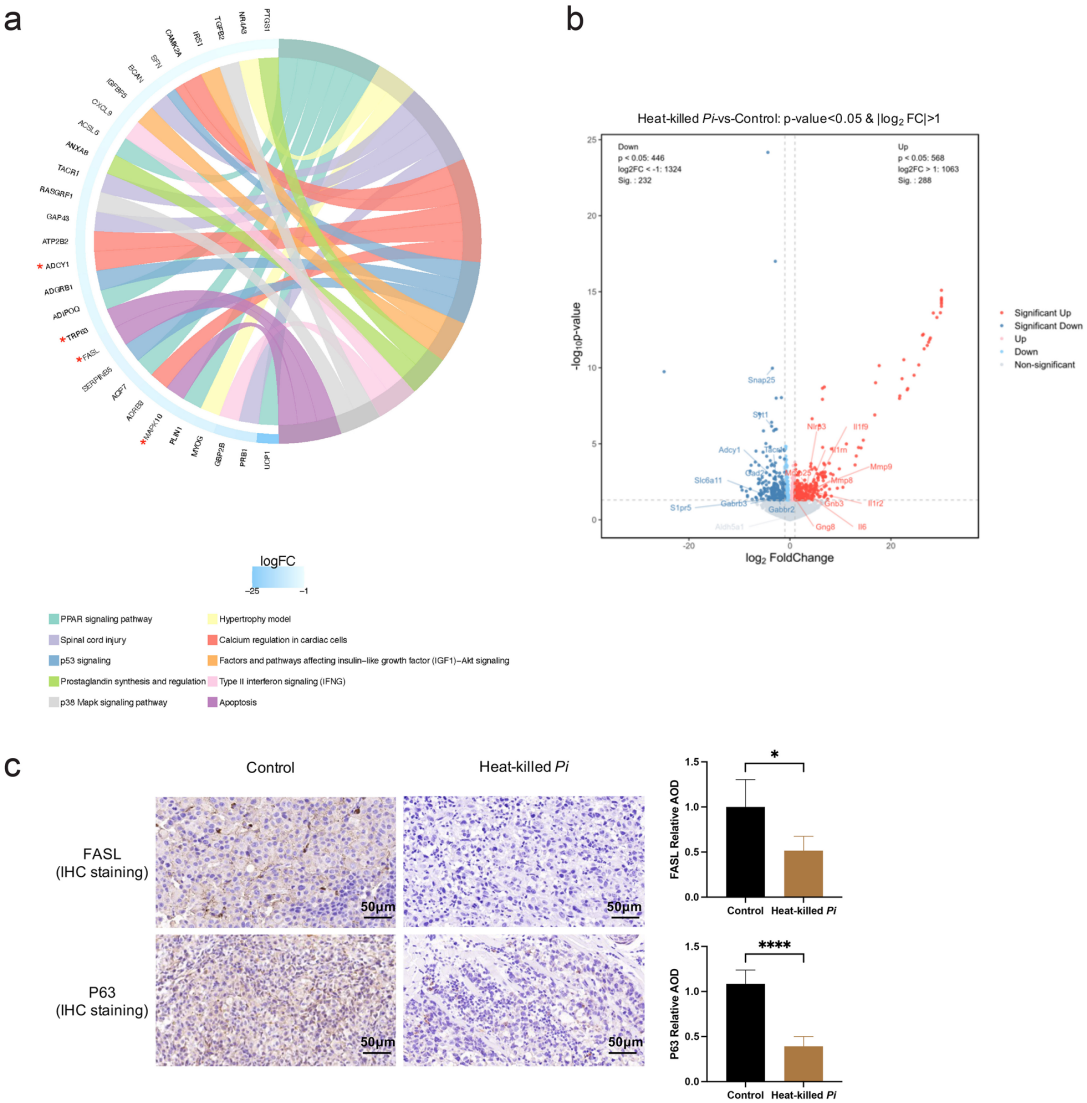
Effects of heat-killed *P*. *intermedia* on the transcriptomics of tumor suppressors in transplanted tumor model mice. **a** Chord diagrams of downregulated genes and pathways. Red asterisks indicate classic tumor suppressor genes. **b** Volcano plot demonstrating the differentially expressed genes in the heat-killed *P*. *intermedia* group compared with the control group. The genes with significantly increased or decreased levels are colored in red or blue, respectively (Wald test, *p* value < 0.05, and fold change > 2). **c** Representative images of FASL and P63 IHC staining. The data are shown as the mean ± SD and were analyzed by *t* test. *P < 0.05, ****P < 0.0001

Transcriptomic profiling was performed to gain insights into the overall alteration of gene expression induced by heat-killed *P*. *intermedia* in OSCC tissues. The results of transcriptomic analysis identified 520 genes with differential expression (fold change > 2) (Additional file 2: Table S1 and Additional file 1: Fig S2) between the control group and heat-killed *P*. *intermedia* group (Additional file 1: Fig S2 and Additional file 2: Table S1). Strikingly, a considerable number of downregulated genes are classic and putative tumor suppressor genes (Additional file 2: Table S1). A database was used for pathway analysis of differentially expressed protein-coding genes. According to enrichment analysis, we found that tumor-associated inflammatory pathways, such as the IL-17, TNF, NF-κB signaling pathways and neutrophil extracellular trap formation-associated pathways, were markedly activated (Fig. 7a, b, Additional file 1: Fig S3). However, GABAergic synapses and glutamatergic synapses were markedly suppressed in the heat-killed *P*. *intermedia* group (Fig. 8a). Moreover, tumor suppressor-associated pathways such as P53 signaling were extensively inhibited (Fig. 9a). Using GSEA, we observed a significant decrease in GABAergic synapses (Fig. 8b), glutamatergic synapses (Fig. 8c), the gamma-aminobutyric acid (GABA) signaling pathway (Fig. 8d), neuroactive ligand‒receptor interactions (Fig. 8e), the synaptic vesicle cycle (Fig. 8f), and GABA synthesis, release, reuptake and degradation (Fig. 8g), accompanied by the downregulated expression of molecules in the GABA family, including GABBR2, GABRB3, SLC6A11, and GAD2 (Fig. 8h–i).

### Heat-killed *P*. *intermedia* altered the protein expression of inflammatory cytokines and tumor suppressors in mouse transplanted tumors

Based on the transcriptomic results and the key role of inflammation in cancer progression, we assessed the protein expression levels of several tumor suppressors and crucial inflammatory cytokines in a mouse transplanted tumor model of OSCC by using IHC staining. We found that the expression levels of crucial molecules in the IL-17 signaling pathway, including IL-17A, IL-6, and IL-23, were significantly enhanced in the heat-killed *P*. *intermedia* group compared with the control group (Fig. 7d), while the expression levels of tumor suppressors, including GABBR2 (Fig. 8j), FASL and P63 (Fig. 9c), in the heat-killed *P*. *intermedia* groups were significantly lower than those in the control group. These results suggested that heat-killed *P*. *intermedia* could regulate immunoinflammatory and tumor suppression-related signaling pathways.

### Heat-killed *P*. *intermedia* increased the number of M2 macrophages in mouse transplanted tumors

**Fig. 10:**
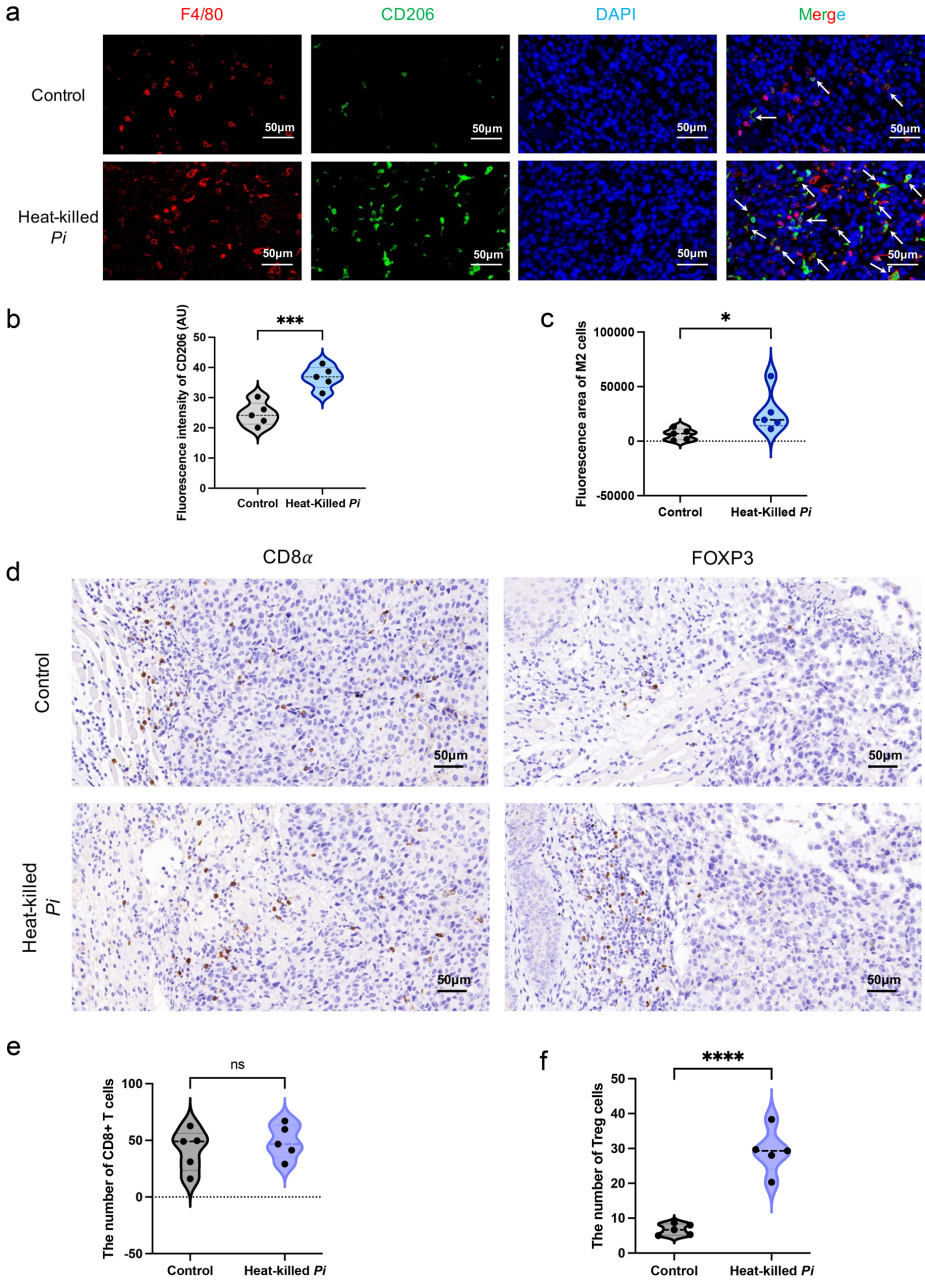
Heat-killed *P*. *intermedia* significantly increased the proportions of M2 macrophages and FOXP3 + Treg cells in mouse transplanted tumors. **a** Representative images of F4/80 (red) and CD206 (green) immunofluorescence staining. White arrows indicate the colocalization of F4/80 and CD206 in the same cell. **b** Fluorescence intensity difference of CD206 between the control and heat-killed *P*. *intermedia* groups. **c** Fluorescence area difference of CD206 between both groups. **d** Representative images of CD8α and FOXP3 IHC staining. **e** The number difference of CD8 + T cells between both groups. **f** The number difference of FOXP3 + Treg cells between both groups. The data are shown as the mean ± SD and were analyzed by *t* test. *P < 0.05, ***P < 0.001, ****P < 0.0001. ns, not significant

M2 macrophages in the TME play a vital role in promoting tumor progression [16]. Therefore, we examined the effects of heat-killed *P*. *intermedia* on M2 macrophage numbers in the OSCC tissues of in situ transplanted tumors in immune-competent mice by using immunofluorescence staining. Our results indicated that the fluorescence intensity and fluorescence area of M2 macrophages (F4/80^+^, CD206^+^) were significantly enhanced in the heat-killed *P*. *intermedia* group compared with the control group (Fig. 10a–c). Therefore, these results suggested that heat-killed *P*. *intermedia* could increase M2 macrophage numbers in OSCC tissues, further exacerbating the immunosuppression in the TME.

### Heat-killed *P*. *intermedia* increased the proportion of Tregs in mouse transplanted tumors

**Fig. 11:**
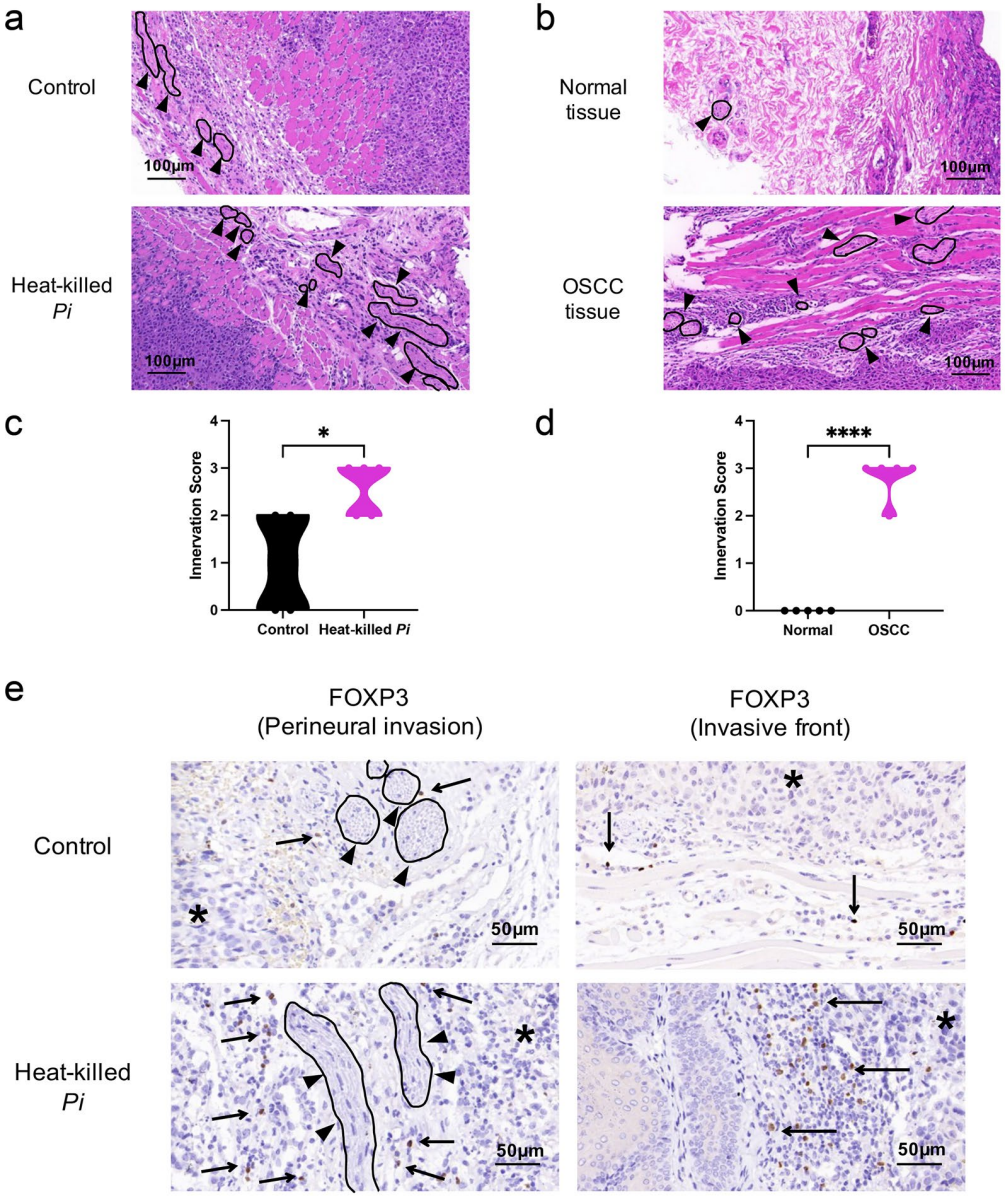
Heat-killed *P*. *intermedia* increased tumor nerve density and perineural invasion. **a** Nerve density in OSCC tissues of mouse transplanted tumors. **b** Representative images of nerve density in normal and OSCC human tissues. **c** Innervation score difference between the control and heat-killed *P*. *intermedia* groups. **d** Innervation score difference between normal and OSCC tissues of humans. **e** FOXP3 + Tregs in perineural areas around the invasive fronts of OSCC can be seen in IHC staining images. Black circles and closed arrowheads show the neural structures. Black asterisks and arrows represent the main tumors and Tregs at tumor invasive fronts, respectively. The data are shown as the mean ± SD and were analyzed by *t* test. *P < 0.05, ****P < 0.0001

To evaluate the effects of heat-killed *P*. *intermedia* on T cells in mouse transplanted tumors, we investigated CD8 + T cells and FOXP3 + Tregs in OSCC tissues by IHC staining. The numbers of CD8 + and FOXP3 + cells were determined with image analyses of immunohistochemistry (Fig. 10d). Our results indicated that the difference in CD8 + T-cell infiltration in OSCC tissues was not statistically significant between the heat-killed *P*. *intermedia* and control groups, while heat-killed *P*. *intermedia* significantly increased the number of Tregs in OSCC tissues (Fig. 10e, f). Intriguingly, our H&E staining results showed that nerve density in the stroma surrounding the tumor markedly increased (Fig. 11a–d), which probably indicates the degree of malignancy [17]. IHC staining indicated that FOXP3 + Tregs mostly accumulated in the invasive fronts of OSCC tissues but were sporadically distributed throughout the tumor. Notably, FOXP3-positive Tregs could be found in perineural areas around the invasive fronts of OSCC (Fig. 11e).

## Discussion

**Fig. 12:**
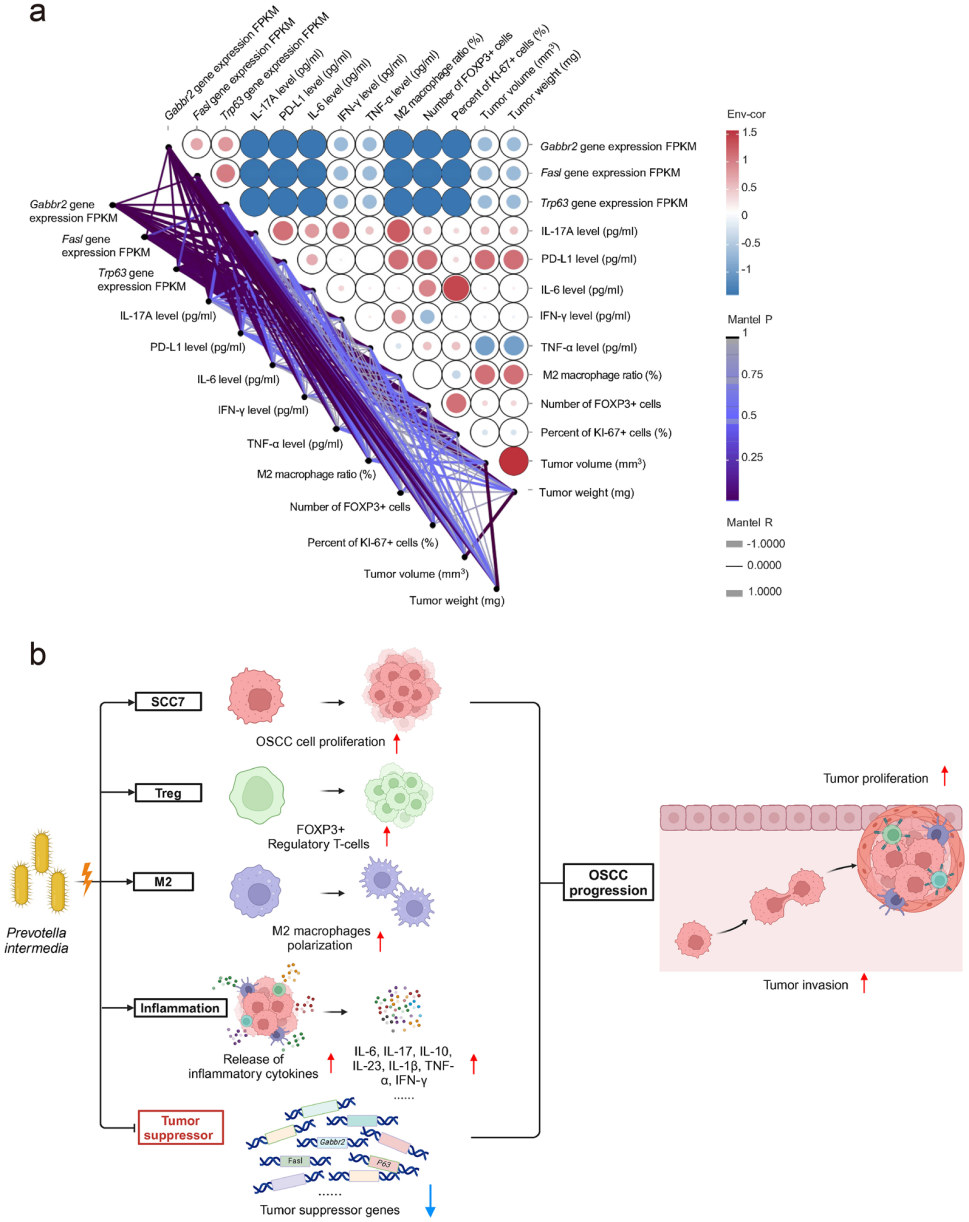
**a** Correlation analysis between the gene expression of three tumor suppressors and tumor-associated factors. Pairwise comparisons of various factors are shown, with a color gradient denoting Spearman’s correlation coefficients. Red and blue nodes denote positive and negative correlations, respectively. Line width corresponds to Mantel’s r statistic for the corresponding distance correlations, and line color denotes statistical significance. **b** Schematic of *P*. *intermedia*-induced OSCC progression showing the effects of heat-killed *P*. *intermedia* on the TME and tumor suppressor genes

Growing evidence indicates that *P*. *intermedia* and other pathogens in the tumor-associated microbiota are specific risk factors for the development and progression of a variety of cancers, including OSCC [3, 5, 7–9, 18]. In this work, we found that *P*. *intermedia* was highly abundant in the tumor tissues of patients with OSCC. Subsequently, we used heat-killed *P*. *intermedia* to treat SCC7 cells in vitro and confirmed that heat-killed* P*. *intermedia* increased SCC7 cell proliferation and invasion and enhanced proinflammatory cytokine release. We further injected heat-killed *P*. *intermedia* into OSCC tissues of transplanted tumor model mice. The in vivo results indicated that *P*. *intermedia* accelerated tumor growth, promoted muscle and perineural invasion of OSCC, elevated serum levels of proinflammatory cytokines, upregulated the expression of proinflammatory cytokines in OSCC tissues, augmented the infiltration of M2 macrophages and Tregs, activated the expression of key molecules in the IL-17A signaling pathway, and inhibited the GABA signaling pathway and an array of tumor suppressor genes in tumor tissues. Our results suggested that heat-killed *P*. *intermedia* could promote OSCC progression by aggravating the inflammatory, immunosuppressive microenvironment while extensively inhibiting tumor suppressor genes (Fig. 12b). In summary, an immunosuppressive microenvironment facilitating OSCC development was shaped by the participation of *P*. *intermedia*.

The evidence suggests that inflammatory cells and cytokines in the tissues that surround tumors increase the risk of chronic, inflammatory conditions progressing to cancer [19]. The microbiota plays a crucial role in cancer by shaping immune system development, immune responses, and metabolism. Microbial dysbiosis can weaken the oral mucosal epithelial barrier, which is conducive to bacterial translocation and macrophage activation, forming protumor chronic inflammation. Translocated symbiotic bacteria trigger the release of protumor inflammatory cytokines, including IL-6, IL-1β, and IL-23, from myeloid cells, which drives the expansion and activation of Th17 cells [20]. A recent study revealed that *C. albicans* promoted OSCC development via the IL-17A/IL-17RA-macrophage axis, and the tumor-associated macrophages (TAMs) attracted by *C. albicans* infection polarized into the M2 phenotype with high expression of PD-L1 [11]. Another study demonstrated that periodontitis-associated oral microbiota could activate IL-17-positive δγ T cells and promote OSCC development and that activated δγT cells were necessary for the IL-17/signal transducer and promoted M2 macrophage infiltration in OSCC proliferation [21]. Researchers have reported that *Prevotella* activates TLR2, leading to the production of Th17-polarizing cytokines, including IL-23 and IL-1. *Prevotella* stimulates epithelial cells to produce IL-8 and IL-6, which can promote mucosal Th17 immune responses and neutrophil recruitment [22]. It has been confirmed by in vitro experiments that *Prevotella* exhibits an enhanced capacity to induce the production of inflammatory mediators (IL-6, IL-8, and TNFα) and enhance neutrophil infiltration [23, 24]. Consistent with these observations, our results indicated that heat-killed *P*. *intermedia* increased the expression levels of IL-17, IL-6, IL-23, IL-10, IL-1β, TNF-α, and PD-L1, induced the release and accumulation of inflammatory cytokines in serum and tumor tissues, and promoted cell proliferation and tumor growth in OSCC. Based on our and other studies, it is suggested that *P*. *intermedia* may accelerate OSCC progression by enhancing chronic inflammatory responses in the TME.

Interestingly, our results of KEGG and GSEA enrichment analyses indicated that both GABAergic synapses and glutamatergic synapses markedly decreased in OSCC tissues after treatment with heat-killed *P*. *intermedia*. Notably, GABA is an important inhibitory neurotransmitter in the body, and emerging data suggest that neurotransmitters can affect immune cells in the TME to regulate tumor progression [25, 26]. Recent studies have confirmed that GABA and GABAB receptors, as potential tumor suppressors, have clear antitumor activity against liver cancer, pancreatic cancer, bile duct cancer, and colorectal cancer [25–30]. In other words, the GABAergic signaling system could reflect antitumor activity by modulating cancer cells and the inflammatory response. Moreover, there is evidence that GABA or GABAergic activation induces autophagy and promotes phagosomal maturation and host antimicrobial responses to intracellular bacterial infections [31]. In our present study, based on the transcriptomic analysis of OSCC tissues, GABAergic signaling was significantly downregulated overall following treatment with heat-killed *P*. *intermedia*. The expression of GABBR2, GABRB3, GABA-transaminase (ABAT), glutamate decarboxylase 2 (GAD2), and Slc6a11 (GAT3) was clearly suppressed in heat-killed *P*. *intermedia*-treated tumor tissues. In summary, our results indicated that the downregulation of the GABAergic pathway may block antitumor immunity and tumor suppression, resulting in OSCC progression.

Generally, the molecular pathogenesis of various cancers, including OSCC, is the result of tumor suppressor inactivation. Our results indicated that the expression levels of an array of classic and putative tumor suppressor genes, such as *P63*, *Fasl*, *Gabbr2*, and *Gzma* (granzyme A), were extensively diminished in mouse transplanted tumors following heat-killed *P*. *intermedia* treatment. Previous studies showed that oral pathogens such as *P. gingivalis* and *F. nucleatum* could accelerate the progression of OSCC by reducing the expression level of the p53 tumor suppressor [32, 33]. Additionally, we verified that heat-killed *P*. *intermedia* inhibited the protein expression of crucial molecules, including GABBR2, P63, and FASL. In summary, *P*. *intermedia* could augment OSCC proliferation by inactivating tumor suppressors.

Nutrient and metabolite alterations in the TME affect tumor-immune interactions. A previous study established that supplementation with intratumoral glutamine inhibited tumor growth by augmenting antitumor immune responses [34]. In contrast, M2 macrophages and Tregs can act as immunological barriers against antitumor immune responses [35]. A study using murine melanoma and lung cancer models indicated that an impaired antitumor immune response is positively correlated with Treg accumulation in the tumor bed, which is reversed after Treg reduction [36]. Moreover, another study has suggested that the overexpression of CXCL1 and CXCR2 has a positive effect on tumor growth by recruiting Tregs [37]. Furthermore, *P. gingivalis* promotes OSCC progression through activation of the CXCL2/CXCR2 axis in the TME [38]. Intratumoral *F. nucleatum* promotes pancreatic cancer progression through autocrine and paracrine mechanisms of the CXCL1-CXCR2 axis [39]. Consistently, under the stimulation of heat-killed *P*. *intermedia* in our study, the glutamatergic system was downregulated, while the expression levels of CXCL1, CXCL2, and CXCR2 increased and Tregs accumulated in tumor tissues, resulting in tumor progression. Altogether, such cross-talk among the nervous system, immune system and TME both systemically and locally regulates protumor inflammation and anticancer immunity [40].

Additionally, macrophages also play an important role in tumor immunity. When macrophages are recruited into the TME, they are extremely heterogeneous and plastic. Macrophage polarization depends on multiple processes, including interactions with other leucocytes and systemic factors such as Tregs, bacterial lipopolysaccharide, and interleukins [41]. TAMs mainly exhibit an M2-like phenotype and stimulate tumor growth by promoting tumor immunosuppression. Polarization to M2 macrophages can be achieved by Tregs [42]. We found that heat-killed *P*. *intermedia* induced M2 macrophage polarization, promoting the progression of OSCC.

## Conclusion

In conclusion, we confirmed a novel association between intratumoral *P*. *intermedia* and OSCC progression and elucidated that intratumoral *P*. *intermedia* is a risk factor that promotes the inflammatory microenvironment, downregulates tumor suppressors, and facilitates tumor cell proliferation. Our data offer a new and potential way to target *P*. *intermedia* and other oral pathogens for the treatment of OSCC in the future.

### Supplementary Information

**Additional file 1: Figure S1.** 16S rRNA partial sequence of *P*. *intermedia* in the NCBI database. **Figure S2.** The number of differentially expressed genes induced by heat-killed *P*. *intermedia* in the transplanted OSCC tumors of mice by transcriptomic analyses. **Figure S3.** Chord diagrams showing the effect of heat-killed *P*. *intermedi*a on the transcriptomics of differential genes and pathways.

**Additional file 2: Table S1.** Heat-killed *P*. *intermedia*-induced differentially expressed genes in transplanted OSCC tumormodel mice. (Classic and putative tumorsuppressor genes are in red and orange, respectively.)

## Data Availability

The data that support the findings of this study are available from the corresponding author upon reasonable request.

## Abbreviations

ANOVA: Analysis of variance
AOD: Average optical density
ATCC: American type culture collection
BHI: Brain heart infusion
CCK-8: Cell counting kit‐8
ELISA: Enzyme-linked immunosorbent assay
FBS: Fetal bovine serum
FISH: Fluorescent in situ hybridization
GABA: Gamma-aminobutyric acid
GSEA: Gene set enrichment analysis
HE: Hematoxylin–eosin staining
MMP-9: Matrix metalloproteinase-9
MOI: Multiplicity of infection
OD: Optical density
OSCC: Oral squamous cell carcinoma
*P*. *intermedia*: *Prevotella intermedia*
POI: Pattern of invasion
SEM: Scanning electron microscope
TAMs: Tumor-associated macrophages
TEM: Transmission electron microscope
TME: Tumor microenvironment
Tregs: Regulatory T cells
