# Supplementary material for: Heat-killed Prevotella intermedia promotes the progression of oral squamous cell carcinoma by inhibiting the expression of tumor suppressors and affecting the tumor microenvironment
Source: Exp Hematol Oncol. 2024 Mar 21;13:33. doi: 10.1186/s40164-024-00500-y (PMC10956211; doi:10.1186/s40164-024-00500-y)
Supplement: Supplementary file 1 — Additional file 1: Figure S1. 16S rRNA partial sequence of P. intermedia in the NCBI database. Figure S2. The number of differentially expressed genes induced by heat-killed P. intermedia in the transplanted OSCC tumors of mice by transcriptomic analyses. Figure S3. Chord diagrams showing the effect of heat-killed P. intermedia on the transcriptomics of differential genes and pathways. [file 40164_2024_500_MOESM1_ESM.docx]

**Intratumoral *Prevotella intermedia* promotes the progression of oral squamous cell carcinoma by inhibiting the expression of tumor suppressors and affecting the tumor microenvironment**

Yifan Zhou, Yao Qin, Jingjing Ma, Zhiyuan Li, Weiwei Heng, Lei Zhang, Hong Liu, Ruowei Li, Miaomiao Zhang, Qiao Peng, Pei Ye, Ning Duan, Ting Liu*, Wenmei Wang*, Xiang Wang*

**Supplementary information**

**Supplementary Figure S1.** 16S rRNA partial sequence of *P*. *intermedia* in *the* NCBI database.

**Supplementary Figure S2.** The number of differentially expressed genesinduced by heat-killed *P*. *intermedia* in the transplanted OSCC tumors of mice by transcriptomic analyses.

**Supplementary Figure S3.** Chord diagrams showing the effectof heat-killed *P*. *intermedia* on the transcriptomics of differential genes and pathways.

**Supplementary Table S1. Heat-killed *P*. *intermedia-*induced differentially expressed genes in transplanted OSCC tumormodel mice. (**Classic and putative tumorsuppressor genes are in red and orange, respectively.)

**Supplementary Figure S1. 16S rRNA partial sequence of *P*. *intermedia* in the NCBI database.** In our present study, the16S rRNA-targeted oligonucleotide probe (RNAscope™ Probe- B-*P*.*intermedia*-16SrRNA-C1) used for *Prevotella intermedia* was designed and developed based on the sequence in the NCBI database, the specificity of which can be confirmed by NCBI database searches and RNAscope studies.

**Supplementary Figure S2.** The number of differentially expressed genesinduced by heat-killed *P*. *intermedia* in the transplanted OSCC tumors of mice by transcriptomic analyses.


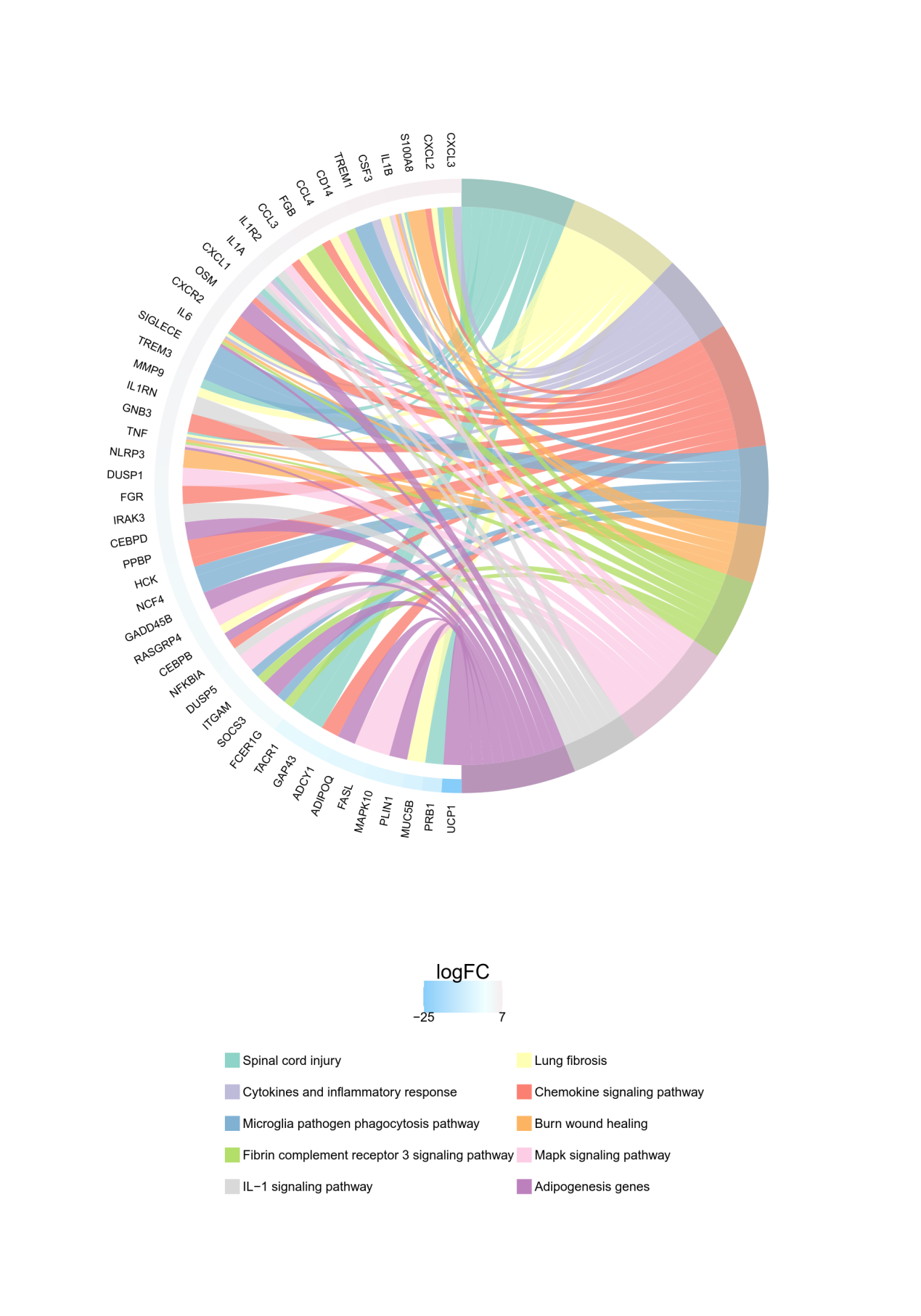


**Supplementary Figure S3.** Chord diagrams showing the effectof heat-killed *P*. *intermedia* on the transcriptomics of differential genes and pathways.
